# Vasculitis and Ischemic Stroke in Lyme Neuroborreliosis—Interventional Management Approach and Literature Review

**DOI:** 10.3390/brainsci13101388

**Published:** 2023-09-29

**Authors:** Burak Han Akkurt, Hermann Kraehling, Nabila Gala Nacul, Mohamed Elsharkawy, Antje Schmidt-Pogoda, Jens Minnerup, Christian Paul Stracke, Wolfram Schwindt

**Affiliations:** 1 Department of Radiology, University Hospital Muenser, Westfalian Wilhelms-University Muenster, Albert-Schweitzer-Campus 1, 48149 Muenster, Germany; 2 Department of Interventional Neuroradiology, Westfalian Wilhelms-University Muenster and University Hospital Muenster, Albert-Schweitzer-Campus 1, 48149 Muenster, Germany; mohamed.elsharkawy@krupp-krankenhaus.de (M.E.); christianpaul.stracke@ukmuenster.de (C.P.S.);; 3 Department of Neurology, Westfalian Wilhelms-University Muenster and University Hospital Muenster, Albert-Schweitzer-Campus 1, 48149 Muenster, Germany; 4 Clinic and Policlinic for Diagnostic and Interventional Neuroradiology, University Hospital Hamburg-Eppendorf, Martinistraße 52, 20246 Hamburg, Germany

**Keywords:** Lyme neuroborreliosis, vasculitis, ischemic stroke, endovascular treatment, neuroradiological intervention, vasospasmolysis, ballon-angioplasty, intracranial stenting, coated stents

## Abstract

Objective: In rare cases, Lyme neuroborreliosis (LNB) can induce cerebral vasculitis leading to severe stenosis of the cerebral vasculature and consecutive ischemia. Therapy is based on anti-biotic treatment of the tick-borne disease, whereas interventional therapeutic options have not been assessed yet. Material and Methods: We report on a patient with LNB and concomitant stenoses and progressive and fatal vasculitis of the cerebral vessels despite all therapeutic efforts by the departments of neurology and interventional neuroradiology. In this context, we also conducted a literature review on endovascular treatment of LNB-associated cerebral ischemia. Results: A 52-year-old female presented with transient neglect and psychomotor slowdown (initial NIHSS = 0). MRI and serology led to the diagnosis of basal meningitis due to LNB with vasculitis of cerebral arteries. Despite immediate treatment with antibiotics and steroids, neurologic deterioration (NIHSS 8) led to an emergency angiography on day 2 after admission. Hemodynamically relevant stenoses of the MCA were treated via spasmolysis and PTA, leading to almost complete neurological recovery. Despite intensified medical treatment, the vasculitis progressed and could only be transiently ameliorated via repetitive spasmolysis. On day 19, she again presented with significant neurologic deterioration (NIHSS 9), and PTA and stenting of the nearly occluded MCA were performed with a patent vessel, initially without hemorrhagic complications. Despite all therapeutic efforts and preserved stent perfusion, vasculitis worsened and the concurrent occurrence of subdural hemorrhage led to the death of the patient. Conclusion: Neuroradiological interventions, i.e., spasmolysis, PTA, and, if necessary, stenting, can and should be considered in cases of LNB-induced vasculitis and stroke that are refractory to best medical treatment alone. Key point: Neuroradiological interventions can be considered in patients with vascular complications of Lyme neuroborreliosis as an additional extension of the primary drug therapy.

## 1. Introduction

Lyme neuroborreliosis (LNB) is an infectious, tick-borne disease of the central nervous system caused by the spirochete *Borrelia burgdorferi*. Borreliosis typically presents with erythema migrans, but the second most common manifestation is neuroborreliosis [1]. The incidence of LNB is up to 2.6 cases per 100,000 individuals [2,3]. Acute neuroborreliosis usually comes with a triad of neurological symptoms: meningitis, cranial neuritis, and radiculoneuritis [1].

However, in rare cases, neuroborreliosis is associated with cerebral vasculitis and can induce cerebrovascular events such as transient ischemic attacks (TIA), intracranial hemorrhage (ICH) or ischemic stroke [4,5]. The guideline-conform treatment of LNB is based on a 2- to 3-week course of antibiotic therapy depending on the onset, mostly with intravenous ceftriaxone or oral doxycycline [6,7].

Here, we present a patient with LNB-associated vasculitis of the cerebral arteries resulting in vasospasms and stenoses that were additionally treated neurointerventionally.

## 2. Clinical Case

### 2.1. Patient

A 52-year-old woman presented with acute onset of neglect and neuropsychological deterioration after an initial “nervous breakdown” on the previous day. Neurological examination showed prolonged psychomotor slowdown; the neglect was not verified and an initial National Institutes of Health Stroke Scale (NIHSS) of 0 was noted. The medical history of the patient showed rheumatoid arthritis and chronic polyarthritis treated with methotrexate and etanercept.

**Figure 1:**
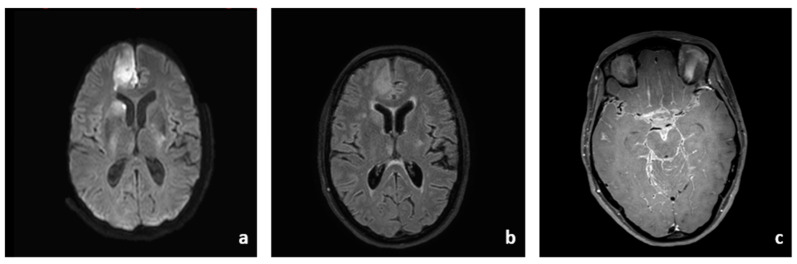
**MRI findings at admission.** Cerebral MRI shows subacute ischemia in the territory of the right ACA and MCA ((**a**): DWI, (**b**): FLAIR). Vessel wall enhancement of the ICA, MCA and ACA indicates vasculitis ((**c**): black blood + gd).

Initial cerebral imaging with magnetic resonance imaging (MRI) showed hyperintense T2w/FLAIR signal of the cerebrospinal fluid and extensive bihemispheric lepto- and pachymeningeal enhancement indicating an inflammatory reaction as well as diffusion restriction in the right anterior cerebral artery (ACA) territory, the head of the caudate nucleus, and the putamen (Figure 1a,b).

Imaging of the brain vessels via Time-of-Flight (ToF)-angiography showed irregularities and stenoses of the right ACA and bilateral middle cerebral arteries (MCA). Contrast-enhanced T1w SPIR (“black blood”) imaging depicted vessel wall enhancement of the right distal internal cerebral artery in the C7 segment and in the M1 segment, indicating vessel wall inflammation (Figure 1c) [8,9].

Neurovascular ultrasound showed bilateral high-grade stenoses in the MCA, high-grade stenosis in the right PCA, low-grade stenosis in the left PCA, low-grade stenosis in the ACA on the left, high-grade stenosis DD occlusion in the right vertebral artery in section V4 with distal refilling, and medium-grade stenosis in the proximal basilar artery.

Routine laboratory tests were unremarkable, despite slightly elevated protein C levels. Initial cerebrospinal fluid (CSF) analysis showed lymphocytic pleocytosis with elevated lactate and decreased glucose levels.

A calculated antibiotic regimen according to national guidelines with ceftriaxone was initiated and supported with dexamethasone [10]. Since tuberculous meningitis was considered a differential diagnosis (potentially facilitated by the anti-rheumatic treatment), the patient additionally received a quadruple therapy with isoniacide, rifampicin, ethambutol, and pyrazinamide. Pathogen diagnostics subsequently yielded positive evidence of IgM and IgG antibodies against *Borrelia burgdorferi*. After supplemental tuberculosis diagnostics, including mycobacteria PCR and staining for acid-fast rods, were negative, Tbc medication was stopped, while ceftriaxone was continued for 24 days.

**Figure 2:**
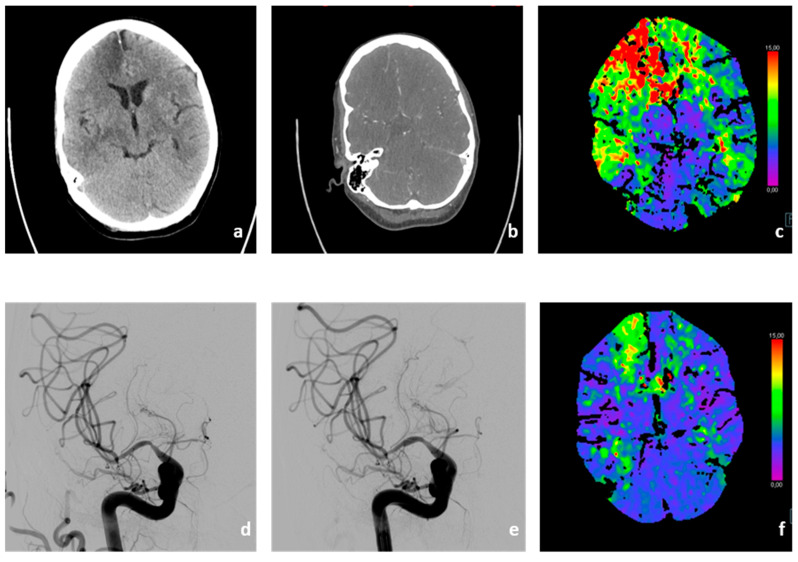
**Progressive deterioration of cerebral perfusion due to vascular stenosis and vasospasms caused by vasculitis.** Progressive right frontal infarct demarcation (**a**) and new onset of stenosis and vasospasms (**b**) leading to massive bihemispheric perfusion delays (**c**). Endovascular vasospasmolysis (**d**) and PTA of high-grade stenoses of the MCA (**e**) shows significant reduction of the aforementioned perfusion delays (**f**).

**Figure 3:**
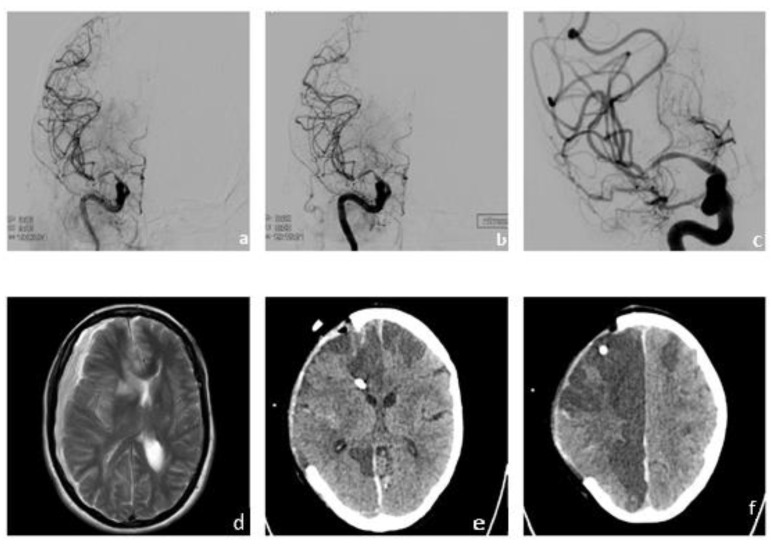
**Follow-up imaging with DSA-series, MRI and CT imaging.** DSA-series showing successful stent implantation in the right MCA with improved vessel diameter/perfusion (**a**–**c**). MRI and CT imaging demonstrating late-onset complications with severe infarct demarcation in both hemispheres and right hemispheric intracerebral hemorrhage as well as subdural hematoma requiring neurosurgical intervention and hemicraniectomy (**d**–**f**).

On the second day after admission, the patient showed severe neurological deterioration with new onset of left-sided hemiparesis, gaze paresis to the left, and tactile neglect (NIHSS 9). Diagnostic cerebral computed tomography showed stenoses of the right MCA and ACA and extensive perfusion deficits next to already demarked infarction in the territory of the right ACA, as previously diagnosed on initial cMRI (Figure 2a–c). An emergency angiography confirmed the hemodynamically relevant stenoses of the right MCA and ACA. Perfusion and stenosis was improved by interdisciplinary consented vasospasmolysis with nimodipine and a percutaneous transluminal angioplasty (PTA) of the right MCA with a remodeling balloon. The proximal stenosis of the MCA was relatively rigid and did not behave as, e.g., vasospasm, due to subarachnoid hemorrhage. The intervention (Figure 2d–e) resulted in almost complete remission of the aforementioned neurological symptoms until day [11], when the patient showed recurrent episodes of initially milder neurological symptoms (e.g., reduced coordination of the left hand) that were repeatedly successfully treated with vasospasmolysis and best medical treatment (altogether seven interventions, Figure 2f). On day 19, however, the neurological symptoms worsened again with severe hemiparesis of the left side and neglect. A multimodal CT showed further worsening of the stenoses and severely reduced cerebral perfusion in the right MCA territory. In interdisciplinary consensus, we performed another PTA of the two high-grade stenoses in the proximal M1 and distal M1 segments of the right MCA after application of 3000 IE heparine. Intraprocedural re-stenosis after PTA necessitated stenting. After administration of 500 mg acetylic salicylic acid (ASA) and initiating an eptifibatide therapy (bolus followed by permanent infusion, body weight adapted) we implanted a self-expanding, fibrin–heparin-coated nitinol microstent (CREDO heal, 4 × 15mm, Acandis, Pforzheim, Germany) in the proximal M1 segment of the right MCA (Figure 3a–c). The perfusion of the right MCA was significantly improved (Figure 3c); angiograms and a flat panel CT showed no complications.

The patient’s neurological symptoms worsened six hours post-intervention with aggravated hemiparesis of the left side. An immediate MRI scan showed a circumscribed intracerebral hemorrhage in the right temporal lobe and a large new subdural hematoma on the right hemisphere with a midline shift to the left and incipient subfalcine and uncal herniation (Figure 3d). The eptifibatid therapy was discontinued and the patient was directly taken to neurosurgery for hemicraniectomy (Figure 3e).

The patient’s MRI and CT images obtained in the following days showed successful evacuation of the SDH, but progressive infarcts in the right MCA territory and bilaterally in the ACA territories (Figure 3f). The implanted stent was patent at all times under platelet inhibition with ASA only.

Due to the poor prognosis and after consultation with the patient’s husband, a palliative therapy concept was pursued from day 24 onwards and she died on day 30 after the initial onset of symptoms.

### 2.2. Neurointerventional Procedures

Due to pronounced stenoses of the cerebral vasculature leading to cerebral perfusion deficits the decision to attempt endovascular treatment was made in an interdisciplinary consensus between the Department of Neurology and the Department for Neuroradiology of the Clinic for Radiology at Muenster University Hospital.

Diagnostic angiography of the cerebral arteries and vasospasmolysis was performed via an arterial transfemoral approach in general anesthesia.

The following materials were used as standard for diagnostic angiography, PTA and vasospasmolysis:

8F sheath, Flowgate balloon catheter + Vertebralis select catheter (Stryker, Freemont, USA), Terumo standard wire (Radiofocus, Tokyo, Japan), Rebar 18 microcatheter (Medtronic, Irvine, USA, 0.021-inch inner diameter), Traxcess 14 microwire (Microvention, Tustin, CA, USA), 2 × 8 mm Neurospeed PTA Ballon Catheter (Acandis, Pforzheim, Germany).

For vasospasmolysis, 2 mg Nimodipin in a total of 40 mL NaCl solution was applied over a period of 20 min for each respective cerebral vascular territory. The success of vasospasmolysis was verified with diagnostic angiography and repeated if necessary. In total, the patient received eight vasospasmolyses over the course of 12 days.

### 2.3. Review of the literature

No approval by the institutional review board was needed for this literature-based review.

We conducted a systematic review of the medical literature to identify published cases of LNB-associated vasculitis and stroke using the online databases of MED-LINE/PubMed between introduction and July 2023.

The search terms were “Lyme neuroborreliosis” OR “neuroborreliosis” OR “borrelio-sis” OR “Borrelia burgdorferi” and one of the following terms “cerebral vasculitis” OR “cerebral vasculopathy” OR “cerebral angiopathy” OR “stroke” OR “ischemia” OR “vasospasm” OR “vasospasmolysis” OR “spasmolysis” OR “stent”.

Inclusion criteria were (1) diagnosis of LNB, (2) associated clinical and/or radiologic evidence of vasculitis and/or stroke, (3) excluded other causes of vasculitis and/or stroke, (4) neurointerventional therapeutic approach.

**Figure 4:**
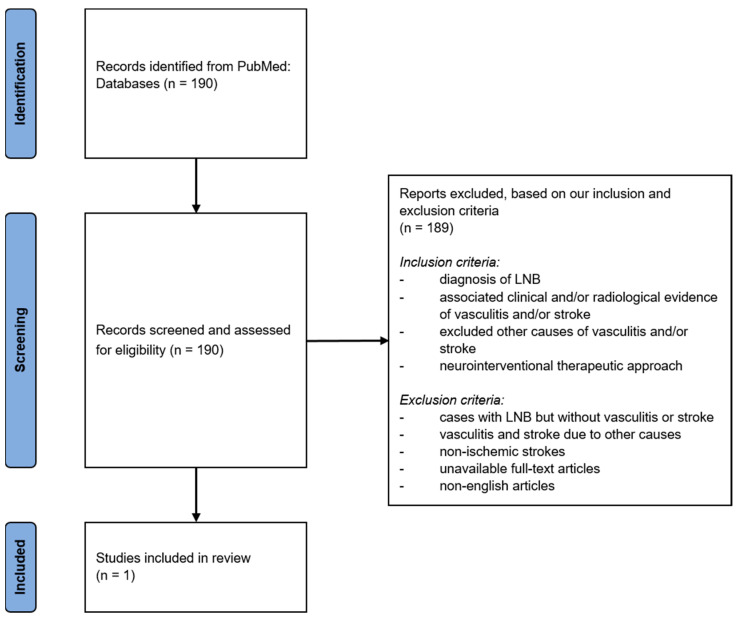
**Flow-chart of the literature review.**

Exclusion criteria were (1) reports on cases with LNB but without final vasculitis or stroke, (2) vasculitis and stroke due to other causes, (3) non-ischemic strokes, (4) unavailable full-text articles, (5) articles written in other languages than English (see Figure 4).

We conducted a review of all articles that met our inclusion and exclusion criteria.

Senior radiology residents with four years of experience in diagnostic neuroradiology independently screened titles and/or abstracts of the studies retrieved by using the aforementioned systematic search plan and extracted data from eligible studies into a standardized form. Discrepancies in the evaluation of the studies were resolved by consensus or by consultation with a senior reviewer.

### 2.4. Results of the Systematic Review

Initial search according to our search strategy showed a total of 190 studies.

Thorough analysis of the available material of each article lead to the exclusion of 189 studies due to a missed topic or not meeting the inclusion criteria.

Our search revealed one study that described acute cerebral ischemia due to large vessel occlusion caused by Lyme neuroborreliosis and treated with endovascular meth-ods.

In this case report, Philipps et al. [11] report on a 6-year-old child with a history of fatigue and headache for 4 weeks, finally resulting in hospitalization because of acute onset of vertigo and impairment of consciousness, speaking and walking. Clinical examination showed, inter alia, anisocoria, dysarthria and tetraplegia in combination with progressive coma (GCS 7, pedNIHSS 32). Anamnestic information revealed a family history of a heterozygous factor V Leiden mutation.

Cerebral imaging was conducted with non-contrast CT showing a hyperdense basilar artery, and MRI confirming basilar occlusion in ToF-angiography with concomitant bilateral pontomesencephalic diffusion restriction in DWI/ADC sequences indicating hyperacute stroke.

Lumbar puncture before MRI revealed the diagnosis of Lyme neuroborreliosis with lymphocytic pleocytosis and elevated antibody index of IgG and IgM for *Borrelia burgdorferi.*

The patient was administered to the angiography unit and received endovascular thrombectomy of the basilar occlusion with an aspiration catheter and Embotrap III stent-retriever together with intra-arterial thrombolysis with 5 mg alteplase.

Antibiotic and anti-inflammatory treatment was started simultaneously after confirmation of diagnosis with cefotaxime IV (3 × 1.3 g) for 16 days and dexamethasone IV (4 mg)/prednisolone PO (30 mg). Furthermore, the patient received tirofiban IV off-label.

Postinterventional imaging showed stable bilateral pontomesencephalic DWI/ADC and FLAIR lesions as well as subtotal reocclusion of the BA and stenosis of the left PCA, that improved gradually in the following weeks without further interventions.

The patient developed well and was finally discharged with a pedNIHSS of 0, mRS 0 under medication with ASA 50 mg/d and prednisolone 15 mg/d [11].

## 3. Discussion

Lyme neuroborreliosis usually presents with the classic triad of meningitis, cranial neuritis and radiculoneuritis but may also have complicating courses including cerebral vessel vasculitis and consequent cerebral ischemia [1]. LNB-associated vasculitis has been reported to occur at rates of 0.3% and usually led to clinically and radiologically proven stroke [2].

Our analysis of the available literature showed that in the majority of the reported cases, a primarily medicinal therapy concept was pursued, while additional neuroradiological interventions have only been reported in a singular case of LNB-associated vasculitis. It is known that neuroradiological interventions such as those presented here have been successfully carried out from the technical aspect in the past in the context of other underlying inflammatory diseases of the cerebral nervous system. In one case, Brisman et al. [12] reported on a patient developing ischemic strokes due to neurosarcoidosis-associated vasculitis causing focal stenosis of the MCA12. A case series published by McKenzie et al. demonstrated technical feasibility of PTA in patients with acute vasculitis [13]. They performed successful PTA in five patients with acute vasculitis of the cerebral vasculature due to different underlying inflammatory diseases. After PTA, all patients showed initial improvement, but clinical status deteriorated in the further course.

The only study so far that describes endovascular treatment of LNB-associated stroke due to large vessel occlusion was conducted in a 6-year-old child with acute basilar occlusion who received intra-arterial thrombolysis and aspiration/stent-retriever-thrombectomy. In this case, presentation in the hyperacute phase of stroke in combination with quick diagnosis and immediate antibiotic and anti-inflammatory treatment together with fast interventional management led to a very good outcome [11]. The literature does not provide a unified explanation for the pathophysiological processes of vasculitis in the setting of infection of the CNS by Borrelia burgdorferi. Therefore, different mechanisms of CNS injury are considered in principle, e.g., a spread of the pathogens along the nerves, as it is also known in the context of radiculitis. Another presumable cause for the development of cerebral vasculitis may be a lymphocytic infiltration of the perivascular space by secondary spread from inflamed meninges to penetrating leptomeningeal and brain parenchymal arteries, causing vascular stenosis and thrombosis [14,15,16]. Topakian et al. report that changes in the vessel walls causing imaging characteristics of infectious vasculitis in LNB are mainly based on inflammatory cells such as lymphocytes and plasma cells infiltrating the vessel walls and the perivascular spaces [2,3]. Moreover, resident inflammatory cells, such as monocytes and macrophages in the perivascular spaces, are thought to trigger a cytokine production which in turn attracts b cells to enter the CNS. Proliferation and production of antibodies amplifies the immune reaction within the CNS, which is believed to cause nerve injuries, analogous to mechanisms of peripheral neural system damages [16]. The distinct (peri-)vascular inflammatory reaction may explain why expanding the vessel during PTA was difficult due to rigid vessel wall structure. On the other hand, acute inflammation can also lead to weakening of the vessel wall, making it more vulnerable for dissection when treated interventionally.

Moreover, bacterial meningitis has been associated with vasospasms of the cerebral arteries and furthermore with permanent neurological deficits and death [17]. Pathological assessment of the vasculature often shows vasospasms rather than vasulitic changes in meningitis-induced stroke [18]. The use of nimodipine has become standard in the prophylaxis and therapy of vasospasms. Recent studies have reported on the application of nimodipine additionally to antibiotic management of LNB with good long-term neurological outcomes [2,19].

To the best of our knowledge, the case presented here is the only report to date of interventional neuroradiological stent treatment in a case of pronounced vasospasms and stenoses of the cerebral vessels due to inflammation in the context of LNB.

Neuroradiological intervention, especially in the setting of florid vasculitis, should always be carefully considered and discussed in detail on an interdisciplinary basis. In the case presented, the clinical symptoms and the vasospasms/persisting stenoses, which could not be treated by other means, forced the interventional steps to be taken. Further passive waiting considering the already manifest clinical symptoms would probably have led to a similarly negative clinical outcome due to the expected occurrence of further ischemia caused by vasospasms and the expected occlusion of the right middle cerebral artery in particular. It is possible that a further acceleration and change in drug therapy could have improved the patient’s outcome, but considering the rapid deterioration of the patient’s condition and the immediate need for neuroradiological intervention as the only short-term therapeutic option, this question remains unanswered.

Furthermore, the respective improvement of the neurological symptoms following a total of eight vasospasmolyses, and especially after the first intervention, showed that these interventions were always able to improve the patient’s condition, even if only for a relatively short time.

The initial therapeutic successes (improvement of the neurological status and the vessel diameter/cerebral perfusion) show that endovascular therapy should always be considered after application of all conservative therapy options and may stabilize the patient’s status until the conservative therapies (antibiosis/anti-inflammation) take effect. Such endovascular therapies involve an increased risk, which is why stenting with consecutive antiplatelet therapy was only used as ultima ratio.

However, the fact that the coated stent remained perfused even without antiplatelet therapy shows the potential of such modern stents.

Nevertheless, such interventions for vasculitides of other etiologies are known and have been successfully performed. In the future, however, the option of neuroradiological intervention should always be discussed in the case of corresponding courses of disease.

## 4. Conclusions

In summary, our study shows the potential of neuroradiological interventions as an ultima ratio alternative attempt in severe vasculitic courses of Lyme neuroborreliosis. Even though the therapy we performed, consisting of vasospasmolysis and stenting of the MCA, only led to short-term improvements in the patient’s symptoms, the performance of such interventions in similar cases of Lyme neuroborreliosis with concomitant vasculitis should not be ruled out per se.

## Data Availability

The datasets used and/or analyzed during the current study are available from the corresponding author on reasonable request.
